# Deep language algorithms predict semantic comprehension from brain activity

**DOI:** 10.1038/s41598-022-20460-9

**Published:** 2022-09-29

**Authors:** Charlotte Caucheteux, Alexandre Gramfort, Jean-Rémi King

**Affiliations:** 1 Meta AI Research, Paris, France; 2 Université Paris-Saclay, Inria, CEA, Palaiseau, France; 3 École normale supérieure, PSL University, CNRS, Paris, France

**Keywords:** Language, Computer science

## Abstract

Deep language algorithms, like GPT-2, have demonstrated remarkable abilities to process text, and now constitute the backbone of automatic translation, summarization and dialogue. However, whether these models encode information that relates to human comprehension still remains controversial. Here, we show that the representations of GPT-2 not only map onto the brain responses to spoken stories, but they also predict the extent to which subjects understand the corresponding narratives. To this end, we analyze 101 subjects recorded with functional Magnetic Resonance Imaging while listening to 70 min of short stories. We then fit a linear mapping model to predict brain activity from GPT-2’s activations. Finally, we show that this mapping reliably correlates ($\mathcal {R}=0.50, p<10^{-15}$) with subjects’ comprehension scores as assessed for each story. This effect peaks in the angular, medial temporal and supra-marginal gyri, and is best accounted for by the long-distance dependencies generated in the deep layers of GPT-2. Overall, this study shows how deep language models help clarify the brain computations underlying language comprehension.

In less than two years, language transformers like GPT-2 have revolutionized the field of natural language processing (NLP). These deep learning architectures are typically trained on very large corpora to complete partially-masked texts, and provide a one-fit-all solution to translation, summarization, and question-answering tasks^1–3^. These advances raise a major question: do these algorithms process language like the human brain? Recent studies suggest that they partially do: the hidden representations of various deep neural networks have shown to linearly predict single-sample fMRI^4–11^, MEG^5,7^, and intracranial responses to spoken and written texts^6,12^.

However, whether these models encode, retrieve and pay attention to information that specifically relates to behavior in general, and to comprehension in particular remains controversial^13–19^. This issue is all-the-more relevant that the behavior of deep language models remains challenged by complex questions, including subject-verb agreement^14,15,17^, causal reasoning^16,19^, story generation, text summarization as well as dialogue and question answering ^20–24^.

To explore the relationship between comprehension and the representations of GPT-2, we compare GPT-2’s activations to the functional Magnetic Resonance Imaging of 101 subjects listening to 70min of seven short stories. We first quantify this similarity with a “brain score” (M)^25,26^. We then evaluate how brain scores systematically vary with – and thus predict – semantic comprehension, as individually assessed by a questionnaire at the end of each story. Finally, by decomposing and manipulating GPT-2’s processes, we identify (1) the brain regions, (2) the levels of representations (phonological, lexical, compositional), and (3) the attentional gating that specifically relates to this prediction.

The alignment identified between behavior, brain activations and the representations of GPT-2 suggest that comprehension relies on a specific computational hierarchy, whereby the auditory cortices integrate information over short time windows, and the fronto-parietal areas combine supra-lexical information over long time windows.

**Figure 1:**
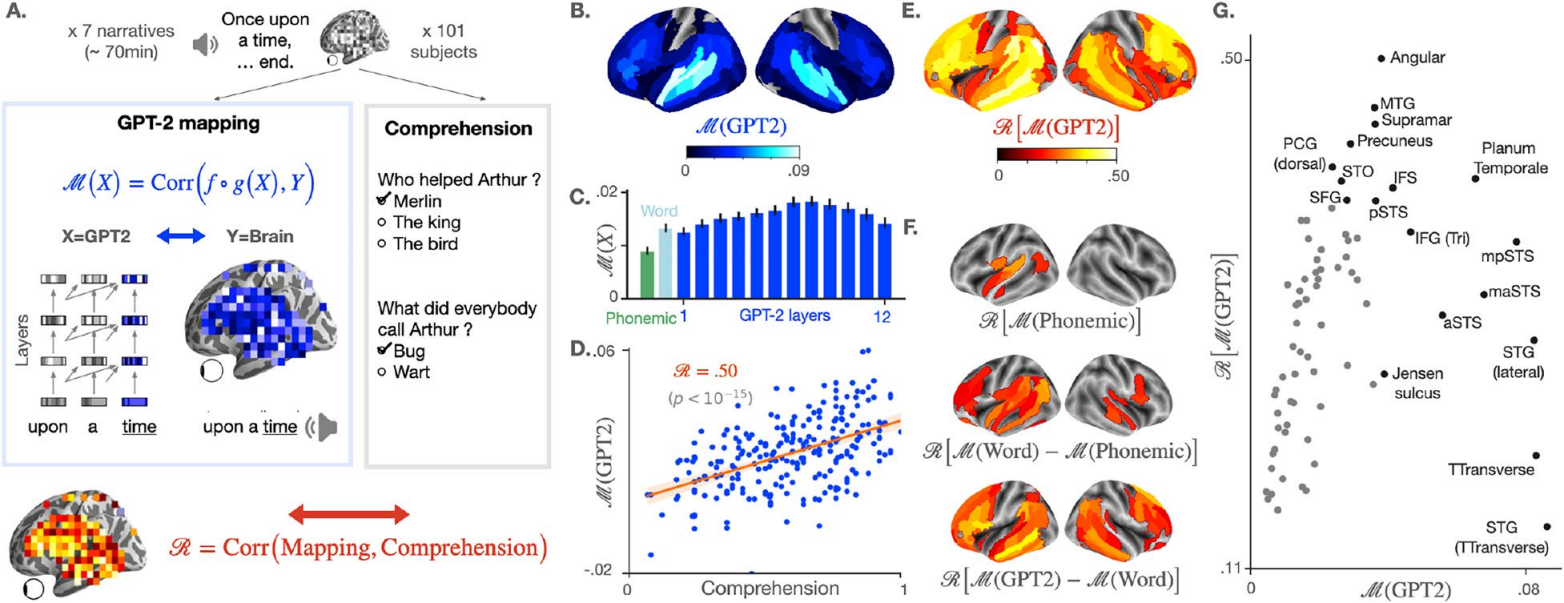
Brain scores and their correlation with comprehension. (**A**) 101 subjects listen to narratives (70 min of unique audio stimulus in total) while their brain signal is recorded using functional MRI. At the end of each story, a questionnaire is submitted to each subject to assess their understanding, and the answers are summarized into a comprehension score specific to each (narrative, subject) pair (grey box). In parallel (blue box on the left), we measure the mapping between the subject’s brain activations and the activations of GPT-2, a deep network trained to predict a word given its past context, both elicited by the same narrative. To this end, a linear spatio-temporal model ($f \circ g$) is fitted to predict the brain activity of one voxel *Y*, given GPT-2 activations *X* as input. The degree of mapping, called “brain score” is defined for each voxel as the Pearson correlation between predicted and actual brain activity on held-out data (blue equation, cf. Methods). Finally, we test the correlation between the comprehension scores of the subjects and their corresponding brain scores using Pearson’s correlation (red equation). A positive correlation means that the representations shared across the brain and GPT-2 are key for the subjects to understand a narrative. (**B**) Brain scores (fMRI predictability) of the activations of the eighth layer of GPT-2. Scores are averaged across subjects, narratives, and voxels within brain regions (142 regions in each hemisphere, following a subdivision of Destrieux Atlas^27^, cf. Supplementary Information A). Only significant regions are displayed, as assessed with a two-sided Wilcoxon test across (subject, narrative) pairs, testing whether the brain score is significantly different from zero (threshold: 0.05). (**C**) Brain scores, averaged across fMRI voxels, for different activation spaces: phonological features (word rate, phoneme rate, phonemes, tone and stress, in green), the non-contextualized word embedding of GPT-2 (“Word”, light blue) and the activations of the contextualized layers of GPT-2 (from layer one to layer twelve, in blue). The error bars refer to the standard error of the mean across (subject, narrative) pairs (*n* = 237). (**D**) Comprehension and GPT-2 brain scores, averaged across voxels, for each (subject, narrative) pair. In red, Pearson’s correlation between the two (denoted $\mathcal {R}$), the corresponding regression line and the 95% confidence interval of the regression coefficient. (**E**) Correlations ($\mathcal {R}$) between comprehension and brain scores over regions of interest. Brain scores are first averaged across voxels within brain regions (similar to **B**), then correlated to the subjects’ comprehension scores. Only significant correlations are displayed (threshold: 0.05). (**F**) Correlation scores ($\mathcal {R}$) between comprehension and the subjects’ brain mapping with phonological features (M(Phonemic) (i), the share of the word-embedding mapping that is not accounted by phonological features $\mathcal {M}(\mathrm {Word}) - \mathcal {M}(\mathrm {Phonemic})$ (ii) and the share of the GPT-2 eighth layer’s mapping not accounted by the word-embedding $\mathcal {M}(\mathrm {GPT2}) - \mathcal {M}(\mathrm {Word})$ (iii). (**G**) Relationship between the average GPT-2-to-brain mapping (eighth layer) per region of interest (similar to **B**), and the corresponding correlation with comprehension ($\mathcal {R}$, similar to **D**). Only regions of the left hemisphere, significant in both (**B**) and (**E**) are displayed. In black, the top ten regions in terms of brain and correlation scores (cf. Supplementary Information A for the acronyms). Significance in (**D**), (**E**) and (**F**) is assessed with Pearson’s *p*-value provided by SciPy^28^. In (**B**), (**E**) and (**F**), *p*-values are corrected for multiple comparison using a False Discovery Rate (Benjamin/Hochberg) over the 2 $\times$ 142 regions of interest.

**Figure 2:**
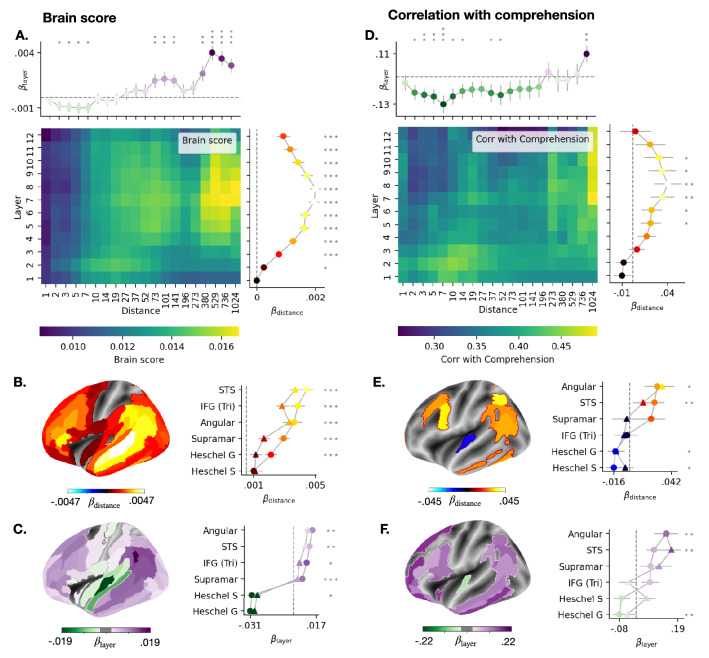
Impact of GPT-2’s attention span on brain scores and comprehension scores. (**A**) The heatmap displays the average (across subjects, stories and voxels) brain scores as a function of attention span (“distance”) and layers. The top line displays the layer coefficients for each attention span (averaged across subjects, stories and voxels). The right line displays the distance coefficient for each layer (averaged across subjects, stories and voxels). The error bars correspond to the Standard Errors of the Mean (SEM) across subject-story pairs. (**B**) Distance coefficients for each brain region (averaged across subjects and stories). Statistical significance is assessed with a Wilcoxon test across subject-story pairs. (**C**) Layer coefficients for each brain region (averaged across subjects and stories). (**D**)–(**F**) Similar as (**A**)–(**C**), but the layer (and distance, respectively) coefficients now assess the relationship between layer (or distance, respectively) and comprehension scores. Statistical significance is assessed using a bootstrapping procedure with 1000 subsamples of subject-story pairs. Error bars are standard deviation across subsamples. For all brain maps, only significant values are displayed ($p<0.05$ after FDR correction across brain regions).

## Results

### GPT-2’s activations linearly map onto fMRI responses to spoken narratives

**Figure 3:**
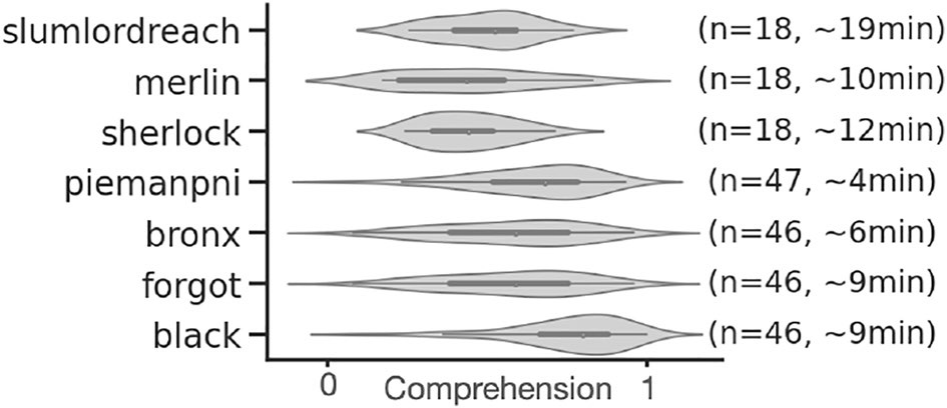
For each of the seven narratives: number of subjects (*n*), distribution of comprehension scores across subjects and length of the narrative.

To assess whether GPT-2 generates similar representations to those of the brain, we analyze the Narratives dataset: 101 subjects listening to seven short stories while their brain activity is recorded with fMRI. Note that subjects do not necessarily listen to the same stories (Fig. 3). First, we evaluate, for each voxel, subject and narrative independently, whether the fMRI responses can be predicted from a linear combination of GPT-2’s activations (Fig. 1A). We summarize the precision of this mapping with a brain score $\mathcal {M}$: i.e. the correlation between the true fMRI responses and the fMRI responses linearly predicted, with cross-validation, from GPT-2’s responses to the same narratives (cf. Methods).

To mitigate the spatial resolution of fMRI and the necessity to correct voxel analyses for multiple comparisons, we here report either 1) the average brain scores across voxels or 2) the average score within each region of interest ($n=314$, following an automatic subdivision of the Destrieux atlas^27^, cf. Supplementary Information A), and correct statistical tests for multiple comparisons across the brain regions. Consistent with previous findings^5,7,29,30^, these brain scores are significant over a distributed and bilateral cortical network, and peak in middle- and superior-temporal gyri and sulci, as well as in the supra-marginal and the infero-frontal cortex^5,7,29^ (Fig. 1B).

By separately analyzing the activations of each layer of GPT-2, we confirm that middle layers best map onto the brain (Fig. 1C), as previously reported^5,7,29^. For clarity, the following analyses focus on the activations extracted from the eighth layer, i.e. the layer with the highest brain score on average across voxels (Fig. 1C). However, the results generalize to other contextual layers of GPT-2 (Supplementary Information E, Supplementary Fig. S4).

### The brain predictions of GPT-2 correlate with semantic comprehension

Does the linear mapping between GPT-2 and the brain reflect a fortunate correspondence^7^? Or, on the contrary, does it reflect similar representations of high-level semantics^8^? To address this issue, we correlate these brain scores to the level of comprehension of the subjects, assessed for each subject-story pair with a questionnaire at the end of each story. On average across all voxels, the correlation between brain scores and comprehension reaches $\mathcal {R}=0.50$ ($p<10^{-15}$, Fig. 1D, as assessed across subject-story pairs with the Pearson’s test provided by SciPy^28^). This correlation is significant across a wide variety of the bilateral temporal, parietal and prefrontal cortices typically linked to language processing (Fig. 1E). Together, these results suggest that the shared representations between GPT-2 and the brain reliably vary with semantic comprehension.

### Low-level processing only partially accounts for the correlation between comprehension and GPT-2’s mapping

Low-level speech representations typically vary with attention^31,32^, and could thus, in turn, influence down-stream comprehension processes. Consequently, one can legitimately wonder whether the correlation between comprehension and GPT-2’s brain mapping is simply driven by variations in low-level auditory processing. To address this issue, we evaluate the predictability of fMRI given low-level phonological features: the word rate, phoneme rate, phonemes, stress and tone of the narrative (cf. Methods). The corresponding brain scores correlate with the subjects’ understanding ($\mathcal {R}=0.17, p<10^{-2}$) but considerably less than the brain scores of GPT-2 ($\Delta \mathcal {R}=0.32$). These low-level correlations with comprehension peak in the left superior temporal cortex (Fig. 1F). Overall, this result suggests that the link between comprehension and GPT-2’s brain mapping may be partially explained by – but not reduced to – the variations of low-level auditory processing.

### High-level representations best predict comprehension

Is the correlation between comprehension and GPT-2’s mapping driven by a *lexical* process and/or by an ability to meaningfully combine words? To tackle this issue, we compare the correlations obtained from GPT-2’s word embedding (i.e. layer 0) to those obtained from GPT-2’s eighth layer, i.e. a contextual embedding. On average across voxels, the correlation with comprehension is 0.12 lower with GPT-2’s word embedding than with its contextual embedding. An analogous analysis, comparing word embedding to phonological features is displayed in Fig. 1F. Strictly lexical effects (word-embedding *versus* phonological) peak in the superior-temporal lobe and in pars triangularis. By contrast, higher-level effects (GPT-2 eighth layer *versus* word-embedding) peak in the superior-frontal, posterior superior-temporal gyrus, in the precuneus and in both the triangular and opercular parts of the inferior frontal gyrus – a network typically associated with high-level language comprehension^7,33–37^. Together, these model comparisons suggest that GPT-2 best predicts how brain responses to speech vary with comprehension.

### Comprehension effects are mainly driven by individuals’ variability

The variability in comprehension scores could result from exogeneous factors (e.g. some stories may be harder to comprehend than others for GPT-2) and/or from endogeneous factors (e.g. some subjects may better understand specific texts because of prior knowledge). To address this issue, we fit a linear mixed model to predict comprehension scores given brain scores, specifying the narrative as a random effect (cf. Supplementary Information B). The fixed effect of brain score (shared across narratives) is highly significant: $\beta =\,0.04, p<10^{-29}$, cf. Supplementary Information B). However, the random effect (slope specific to each single narrative) is not ($\beta <10^{-2}$, $p>\,0.11$). We also replicate the main analysis (Fig. 1D) within each single narrative: the correlation with comprehension reaches 0.76 for the ‘Sherlock’ story and is above 0.40 for every story (cf. Supplementary Information C). Overall, these analyses confirm that the link between GPT-2 and semantic comprehension is best accounted for by an endogeneous factor: i.e. individual differences in comprehension scores.

### Decomposing the brain regions, levels of representation and attention distances underlying comprehension

Can GPT-2 be further decomposed to identify the mechanisms responsible for generating representations that both (i) map with the human brain and (ii) predict subjects’ comprehension? To address this issue, we investigate the links between (1) short- and long-range attentional gating, (2) the depth of the representation and (3) brain and comprehension scores. Specifically, we compute both of these scores for different GPT-2 layer *k*, when restricting their attention span to different distances *d* (i.e. layers $k'\le k$ only access the *d* previous words). By systematically and independently varying *k* and *d*, we can compute $\beta _{\mathrm {distance}}$ and $\beta _{\mathrm {layer}}$: the two coefficients that indicate how brain scores and comprehension scores vary across layers and attentional spans, respectively. Precisely, a positive $\beta _{\mathrm {distance}}$ indicates that scores are sensitive to long-range dependencies. On the contrary, a null $\beta _{\mathrm {distance}}$ indicates that scores are not sensitive to long-range-dependencies. Similarly, a positive $\beta _{\mathrm {layer}}$ indicates that deep layers have better scores than shallow layers, while a negative $\beta _{\mathrm {layer}}$ indicates that shallow layers have better scores than deep layers.

Our results are three-fold. First, both the brain score ($\mathcal {M}$) and the comprehension scores ($\mathcal {R}$) increase with the attention span ($\beta _{\mathrm {distance}}>0$, $p^{M}<10^{-14}$ for brain scores, $p^{R}=\,0.01$ for comprehension scores) as well as with the depth of the representation ($\beta _{\mathrm {layer}}>0$, $p^{M}< 10^{-4}$, $p^{R}=\,0.001$). The gain in scores obtained with attention to distant context is observed even up to the most distant items (e.g. between distance $\approx 1000$ and 300 words: $\Delta R>\,0$, $p^{M}<10^{-4}$, $p^{R}=\,0.02$, Fig. 2A).

Second, the attention span primarily impacts the brain scores and the comprehension scores of the middle layers (difference between layer 8 and layer 12: $\Delta \beta _{\mathrm {distance}}=\,0.001$, $p^{M}<10^{-8}$ for brain scores, $\Delta \beta _{\mathrm {distance}}=\,0.03$, $p^{R}=\,0.005$ for comprehension scores, Fig. 2AD). Interestingly, and to our surprise, restricting the attention span of the first layers improved their ability to predict comprehension (e.g. for the first layer, difference between scores with an attention of 10 words and full attention $\Delta R=\,0.06$, $p=\,0.004$, Fig. 2D). This unexpected result suggests that language transformers could be made more similar to the brain by increasing the attention span as a function of depth.

Finally, brain regions commonly associated with high-level comprehension are better predicted by the deep and contextual representations of the network, and their corresponding brain scores and comprehension scores are relatively strongly modulated by long-distance attention (e.g. in angular gyrus: $\beta _{\mathrm {layer}}= 0.14>0$, $p=\,0.002$, $\beta _{\mathrm {distance}}=\,0.03>0$, $p=\,0.016$ for comprehension scores). On the contrary, low-level acoustic regions are best predicted by the shallow layers of the network, and are, in comparison, little altered by long-distance dependencies (e.g. for the comprehension scores in Heschl gyrus, $\beta _{\mathrm {layer}}=\,-0.076<0$, $p=\,0.004$, $\beta _{\mathrm {distance}}=\,-0.014<0$, $p=\,0.012$).

Overall, our analysis suggests that comprehension depends on a hierarchy of neural representations, whereby the first areas of the language network deploys shallow and short-span attention processes, while the fronto–parietal network relies on compositional and long-span attention processes. Interestingly, our analysis also highlights that shortening the attention span of lower layers makes them more brain-like, and could perhaps thus provide a useful inductive bias to these algorithms.

## Discussion

Our analyses reveal a reliable correlation between story comprehension and the degree to which language transformers like GPT-2 maps onto brain responses to the corresponding story. Furthermore, the systematic comparison, decomposition and manipulation of such language models allow us to decompose (1) the brain regions (2) the level of representation (sub-lexical, lexical, supra-lexical) and (3) the attentional gating (i.e. the short- or long-range retrieval of past stimuli) that relate to the comprehension of complex narratives.

These findings complement prior work on the brain bases of comprehension in three major ways. First, a number of qualitative theories describe how words may be combined into meaningful representations^36–43^. For example, the Memory, Unification and Control model (MUC) distinguishes three types of computations and links them to the temporal lobe, Broca area and the rest of the prefrontal lobe, respectively. Similarly, the extended Argument Dependency Model (eADM) proposes that the ventral and the dorsal streams of the auditory pathway compute time-independent and time-dependent unifications, respectively. Our results support an analogous division of acoustics, lexical and compositional representations in the language areas. However, we reveal a slightly different functional anatomy: the early areas of the language network, located around the auditory cortices, deploy sub-lexical and shallow representations thanks to short attention spans. By contrast, the fronto–parietal network tracks and unifies very distant contexts to current words (Fig. 1F). How these cortical areas communicate with the hippocampus and retrieve words from long-term memory remains an exciting direction for future studies^44^.

Second, several quantitative approaches have been proposed to investigate comprehension, either with “model-free” methods based on inter-subject correlation (e.g.^33,35,45^) or “model-based” methods based on word vectors^46^. For example, Lerner et al. analyzed the fMRI activity of subjects listening to either normal texts or texts scrambled at the word, sentence or paragraph level^33^. While brain activity correlated across subjects in the primary and secondary auditory areas even when the input was heavily scrambled (and thus poorly comprehensible), the bilateral infero-frontal and temporo-parietal cortex only correlated across subjects when sentences and/or paragraphs were not scrambled (and thus comprehensible). Broderick et al. used a similar design to investigate electro-encephalography (EEG) responses to variably scrambled versions of the same story^46^, as well as the EEG responses to speech played in reverse and in noise^47^. Consistently with our results, they showed that the mapping between word embeddings’ and the EEG activity varies with comprehension as manipulated by these various protocols. Our results thus complement these findings by showing (1) the brain regions where GPT-2’s predictions vary with subject’s comprehension, and (2) what type of representations these features relate to: comprehension appears here to depend on a hierarchy of neural representations, whereby the first areas of the language network deploys shallow and short-span-attention processes, while the fronto–parietal network relies on compositional and long-span-attention processes.

Finally, previous analyses have investigated the role of attention in the brain^5,48,49^. We complement these studies by (1) showing that very-long term attention affects brain scores (even above 1,000 words), (2) identifying the brain regions that are sensitive to long vs. short attention spans, and(3) investigating the interactions between attention span, the ability to generate brain-like representations, and one behavioral metric: comprehension.

Interestingly, some regions, like the angular and supramarginal gyri, present a modest brain score and nevertheless strongly predict comprehension. How can one interpret such dissociation? We propose that deep neural networks encode a variety of features, ranging from low- to high-level representations. While some of these features may relate to general language processing (e.g. short-range information about words), others may specifically relate to and thus predict comprehension (e.g. long-range dependencies). In this view, the regions that are best predicted by GPT-2’s representations (e.g. Heschel’s gyrus) need not be identical to those that best predict comprehension (e.g. Angular gyrus). Our ablation studies fit this hypothesis: the auditory cortices are marked by high brain scores but low comprehension scores (Fig. 1G) and indeed appear to encode short-range and shallow representations – i.e. features that presumably only indirectly relate to the comprehension of a narrative (Fig. 2). By contrast, the angular gyrus demonstrates a high comprehension score (Fig. 1G) and indeed appears to encode long-range dependencies and deep representations – i.e. features that presumably relate to the latent structures of narratives, and from which comprehension should depend (Fig. 2).

Overall, the present study suggests that GPT-2 retrieves information that relates to human comprehension, thus strengthening previous works that study the similarities between deep language models and the brain^4–12^. For instance, several studies showed that deep nets’ encoding accuracy correlated with the level of semantic and syntactic information of their activations^11^, as well as their ability to predict a word from context^6,7^. We complement these results and show that the encoding accuracy of GPT-2 correlates with the level of understanding of the subjects, as assessed with comprehension questionnaires. Interestingly, our analysis also highlights that shortening the attention span of the lower layers would make them more brain-like. Thus, these results contribute to revealing remaining functional differences between brains and language models, and could thus help guide the development of modern algorithms^5,50^.

The relationship between GPT-2’s representations and human comprehension remains to be qualified, however. First, we restrict the challenging and composite notion of semantic comprehension to an empirical definition: i.e. the extent to which subjects understand a narrative, as assessed by a questionnaire presented at the end of each story. We acknowledge that comprehension spans a very diverse set of conditions, ranging from scientific writing to newspapers, which are not presently tested.

Second, our results remain solely based on correlations. Supplementary analyses suggest that GPT-2’s brain scores may be partially explained by – but not reduced to – attentional processes (Supplementary Information H). Yet, the factors that causally influence comprehension, such as attention, prior knowledge, working memory capacity, and language complexity are not controlled here and should thus be explicitly examined and manipulated in future work. In particular, it would be interesting to evaluate how working memory capacity, cognitive control, vocabulary, as well as an continuous-monitoring of subjects’ attention separately contribute to the fluctuation of comprehension and specifically account for the link between GPT-2 and the brain. Similarly, the study of inter-individual differences could further help modeling specific cognitive deficits associated with comprehension such as dyspraxia, dyslexia or autistic syndrome. However, such investigation would likely require large amounts of data, and thus a dedicated effort^51^.

Third, we find that the long-distance representations of GPT-2 middle layers specifically account for comprehension in associative cortices, while the short-distance information encoded in the shallow layers account for comprehension in lower-level brain regions. However, what these features actually represent remains largely unknown. Previous studies have shown that language transformers explicitly represent syntactic^14,52^ and semantic features^14^. Similarly, Manning et al. showed that syntactic trees appear to be encoded by the distances between contextualized word embedding^52^. Clarifying the nature of word embeddings remains an important direction to explore (e.g. syntactic vs. semantic^8,11,53,54^.

Finally, although highly significant, and significantly better than alternative models (Supplementary Fig. S3), the brain-scores of GPT-2 are relatively low^5,26,35^. This phenomenon is largely expected: we fit and evaluate the brain mapping at the single-TR single-voxel level and across all brain voxels to avoid selection biases. Nonetheless, these brain scores reach up to 32% of the noise ceiling (Supplementary Information D, Supplementary Fig. S2). This indicates that while GPT-2 may be our best model of language representations in the brain, it remains far from fully capturing those of complex narratives.

The comparison between brains, behavior and deep nets was originally introduced in vision research^55^. The present study strengthens this approach and clarifies the links between GPT-2 and the brain. Specifically, we show that GPT-2’s mapping correlates with comprehension up to $\mathcal {R}=0.50$. This result is both promising and limited: on the one hand, we reveal that the similarity between deep nets and the brain non-trivially relates to a high-level cognitive process. On the other hand, half of the comprehension variability remains unexplained by this algorithm.

This limit is expected: several studies demonstrate that current deep language models fail to capture several aspects critical to comprehension^16,19^: they (i) often fail to generalize beyond the training distribution^56^, (ii) do not perfectly capture deep syntactic structures^14,52^ and (iii) remain relatively poor at summarizing texts, generating stories and answering questions^20–22^. Furthermore, GPT-2 is only trained with textual data and does not situate objects in a grounded environment that would capture their real-world interactions^18,57^. These limits may be temporary, however: the latest models appear to be more robust to out-of-distribution sampling^58^ and trained on multimodal data^59,60^.

Together, these elements suggest that modern language algorithms like GPT-2 offer a promising basis to unravel the brain and computational signatures of comprehension. Vice versa, by highlighting the similarities and remaining differences between deep language models and the brain, our study reinforces the mutual relevance of neuroscience and AI.

## Materials and methods

Our analyses rely on the “Narratives” dataset^61^, composed of the brain signals, recorded using fMRI, of 345 subjects listening to 27 narratives. The dataset is publicly available and the methods were performed in accordance with relevant guidelines and regulations.

### Narratives and comprehension score

Among the 27 stories of the dataset, we selected the seven stories for which subjects were asked to answer a comprehension questionnaire at the end, and for which the answers varied across subjects (more than ten different comprehension scores across subjects), resulting in 70 min of audio stimuli in total, from four to 19 minutes per story (Fig. 3). Questionnaires were either multiple-choice, fill-in-the blank, or open questions (answered with free text) rated by humans^61^. Here, we used the comprehension score computed in the original dataset which was either a proportion of correct answers or the sum of the human ratings, scaled between 0 and 1^61^. It summarizes the comprehension of one subject for one narrative (specific to each (narrative, subject) pair).

### Brain activations

The brain activations of the 101 subjects who listened to the seven selected narratives were recorded using fMRI. As suggested in the original paper^61^, pairs of (subject, narrative) were excluded because of noisy recordings, resulting in 237 pairs in total.

All seven studies used a repetition time (TR) of 1.5 seconds. As stated in the orginal paper^61^, the “Merlin”, “Sherlock”, “Slumlord” and “Reach for the Stars” datasets were collected on a 3T Siemens Magnetom Skyra (Erlangen, Germany) with a 20-channel phased-array head coil using the following acquisition parameters. “Functional BOLD images were acquired in an interleaved fashion using gradient-echo echo-planar imaging (EPI) with an in-plane acceleration factor of 2 using GRAPPA. The full acquisition details are summarized here for simplicity: TR/TE = 1500/28 ms, flip angle = 64 degrees, bandwidth = 1445 Hz/Px, in-plane resolution = 3x3mm, slice thickness $= 4$ mm, matrix size = $64\times 64$, FoV = $192\times 192$ mm, 27 axial slices with roughly full brain coverage and no gap, anterior–posterior phase encoding, prescan normalization, fat suppression. At the beginning of each run, three dummy scans were acquired and discarded by the scanner to allow for signal stabilization.

The “Pie Man (PNI)” (pieman-pni) “Running from the Bronx”(bronx), “I Knew You Were Black” (black) and “The Man Who Forgot Ray Bradbury”(forgot) datasets were collected on the same 3T Siemens Magnetom Prisma with a 64-channel head coil using different acquisition parameters. Functional images were acquired in an interleaved fashion using gradient-echo EPI with a multiband acceleration factor of 3 using blipped CAIPIRINHA and no in-plane acceleration: TR/TE 1500/31 ms, flip angle = 67 degrees, bandwidth = 2480 Hz/Px, in-plane resolution = $2.5\times 2.5$ mm, slice thickness 2.5 mm, matrix size = $96\times 96$, FoV = $240 \times 240$ mm, 48 axial slices with full brain coverage and no gap, anterior–posterior phase encoding, prescan normalization, fat suppression, three dummy scans.”

### GPT-2 activations

GPT-2^1^ is a high-performing neural language model trained to predict a word given its previous context (it does not have access to succeeding words), given millions of examples (e.g Wikipedia texts). It consists of multiple Transformer modules (twelve, each of them called “layer”) stacked on a non-contextual word embedding (a look-up table that outputs a single vector per vocabulary word)^1^. Each layer *k* can be seen as a nonlinear system that takes a sequence of *w* words as input, and outputs a contextual vector of dimension (*w*, *d*), called the “activations” of layer *k* ($d=\,768$). Intermediate layers were shown to better encode syntactic and semantic information than input and output layers^62^, and to better map onto brain activity^5,7^. Here, we show that the *eighth* layer of GPT-2 best predicts brain activity Fig. 1C. We thus select the eighth layer of GPT-2 for our analyses. Our conclusions remain unchanged with other intermediate-to-deep layers of GPT-2 (from $6^{\text{th}}$ to $12^{\text{th}}$ layers).

In practice, the narratives’ transcripts were formatted (replacing special punctuation marks such as “–” and duplicated marks “?.” by dots), tokenized using GPT-2 tokenizer and input to the GPT-2 pretrained model provided by Huggingface^63^. The representation of each token is computed separately using a sliding context window of 1024 tokens. For instance, to compute the representation of the third token of the story, we input GPT-2 with the third, second and first token, and then extract the activations corresponding to the third token. Similarly, to compute the activations of the $1500^{\text{ th}}$ token, we input the model with the word 1500 and the 1023 words before. Overall, the activations of every word $w_k$ are computed by inputting the model with the word $w_k$ and the 1023 previous tokens (at most), and then extracting the activations corresponding to $w_k$. The procedure results in a vector of activations of size (*w*, *d*) with *w* the number of tokens in the story and *d* the dimensionality of the model. There are fewer fMRI scans than words. Thus, the activation vectors between successive fMRI measurements are summed to obtain one vector of size *d* per measurement. To match the fMRI measurements and the GPT-2 vectors over time, we used the speech-to-text correspondences provided in the fMRI dataset^61^.

### Linear mapping between GPT-2 and the brain

For each (subject, narrative) pair, we measure the mapping between (i) the fMRI activations elicited by the narrative and (ii) the activations of GPT-2 (layer eight) elicited by the same narrative. To this end, a linear spatiotemporal model is fitted on a train set to predict the fMRI scans given the GPT-2 activations as input. Then, the mapping is evaluated by computing the Pearson correlation between predicted and actual fMRI scans on a held out set *I*:

1 $$\begin{aligned} {\mathcal {M}}^{(s, w)} : I \mapsto \mathcal {L} \bigg ( f \circ g(X^{(w)})_{i \in I}, (Y^{(s, w)}_i)_{i \in I} \bigg ) \end{aligned}$$

With $f\circ g$ the fitted estimator (g: temporal and f: spatial mappings), $\mathcal {L}$ Pearson’s correlation, $X^{(w)}$ the activations of GPT-2 and $Y^{(s, w)}$ the fMRI scans of subjects *s*, both elicited by the narrative *w*.

In practice, *f* is a $\ell _2$-penalized linear regression, following scikit-learn implementation^64^. The regularization parameter is chosen for each voxel separately using nested cross validation on the train set. Specifically, we use scikit-learn’s RidgeCV estimator with built-in leave-one-sample-out cross-validation, with ten possible regularization parameters log-spaced between $10^{-1}$ and $10^8$, one hyper-parameter being selected for each voxel independently. *g* is a finite impulse response (FIR) model with 5 delays, where each delay sums the activations of GPT-2 input with the words presented between two TRs. For each (subject, narrative) pair, we split the corresponding fMRI time series into five contiguous chunks using scikit-learn cross-validation. The procedure is repeated across the five train (80% of the fMRI scans) and disjoint test folds (20% of the fMRI scans). Pearson correlations are averaged across test folds to obtain a single score per (subject, narrative) pair. This score, denoted $\mathcal {M}(X)$ in Fig. 1A, measures the mapping between the activations space *X* and the brain of one subject, elicited by one narrative.

### Phonological features

To account for low-level speech processing, we computed the alignment (Eq. (1)) between the fMRI brain recordings *Y* and phonological features *X*: the word rate (of dimension $d=\,1$, the number of words per fMRI scan), the phoneme rate ($d=\,1$, the number of phonemes per fMRI scan) and the concatenation of phonemes, stresses and tones of the words in the stimuli (categorical feature, $d=\,117$). The latter phonological features are provided in the original dataset and computed using Gentle^65^. The 117 dimensions are the combination of phonetic categories, stresses and tones. We use 40 English phonemes in the corpus, and 4 possible tones, which results in 40 x 4 = 160 possible categories. Some categories are never pronounced here. If we ignore these categories, this results in 117 categories, and thus 117 dimensions after one-hot encoding.

### Voxel-level and ROI-level analyses

All of the first-level analyses are performed at the voxel level (computation of the mapping scores $\mathcal {M}$ in Eq. (1), in blue in Fig. 1). We then average these effects either (1) within each brain region (Fig. 1B, E, F and G) or (2) across the whole brain (Fig. 1C and D). From these average values, we compute the correlation with comprehension (in red in Fig. 1). This approach mitigates the localization of the effect and the statistical correction for multiple comparisons.

### Significance

Significance was either assessed by using either (i) a second-level Wilcoxon test (two-sided) across subject-narrative pairs, testing whether the mapping (one value per pair) was significantly different from zero (Fig. 1B), or (ii) by using the first-level Pearson *p*-value provided by SciPy^28^ (Fig. 1D–G). In Fig. 1B, E, F, *p*-values were corrected for multiple comparison (2 $\times$ 142 ROIs) using False Discovery Rate (Benjamin/Hochberg)^66^.

## Supplementary Information

Supplementary Information.

## Data Availability

The Narratives dataset^61^ is publicly available on the OpenNeuro (https://openneuro.org/datasets/ds002345/versions/1.1.4) and Datalad platforms (http://datasets.datalad.org/?dir=/labs/hasson/narratives).
