# Supplementary material for: Deep language algorithms predict semantic comprehension from brain activity
Source: Sci Rep. 2022 Sep 29;12:16327. doi: 10.1038/s41598-022-20460-9 (PMC9522791; doi:10.1038/s41598-022-20460-9)
Supplement: Supplementary file 1 — Supplementary Information. [file 41598_2022_20460_MOESM1_ESM.pdf]

Supporting Information (SI)

A. Brain parcellation. In Figure 1B, E, and F, we used a subdivision of the parcellation from Destrieux Atlas (27). Regions with more than 400 vertices were split into smaller regions (so that each regions contains less than 400 vertices). The original parcellation consists of 75 regions per hemisphere. Our custom parcellation consists in 142 regions per hemisphere.

In Figure 1G, we use the original parcellation for simplicity, and the following acronyms:

Acronym	Definition
STG / STS	Superior temporal gyrus / sulcus
aSTS	Anterior STS
maSTS	Mid-anterior STS
mpSTS	Mid-posterior STS
pSTS	Posterior STS
Angular / Supramar	Angular / Supramarginal inferior parietal gyrus
MTG / MTS	Medial temporal gyrus / sulcus
SFG / SFS	Superior frontal gyrus / sulcus
IFG / IFS	Inferior frontal gyrus / sulcus
Tri / Op	Pars triangularis / opercularis (IFG)
TTransverse	Temporal transverse sulcus
PCG	Posterior cingulate gyrus
STO	Temporo-occipital lateral sulcus

B. Mixed-effect model. Not all subjects listened to the same stories. To check that the $\mathcal{R}$ scores (correlation between comprehension and brain mapping) were not driven by the narratives and questionnaires' variability, a linear mixed-effect model was fit to predict the comprehension of a subject given its brain mapping scores, specifying the narrative as a random effect. More precisely, if $\mathcal{M}_{w_i} \in \mathbb{R}$ corresponds to the mapping scores of the ith subject that listened to the story w, and $C_{w_i} \in \mathbb{R}$ refers to the comprehension scores, we estimate the fixed effect parameters $\widetilde{\beta} \in \mathbb{R}$ and $\widetilde{\eta} \in \mathbb{R}$ (shared across narratives), and the random effect parameter $\beta_w \in \mathbb{R}$ and $\eta_w \in \mathbb{R}$ (specific to the narrative w) such that:

$$C_{w_i} = (\widetilde{\beta} + \beta_w) \times \mathcal{M}_{w_i} + (\widetilde{\eta} + \eta_w) + \epsilon_{w_i}$$

with ϵ_{w_i} a vector of i.i.d normal errors with mean 0 and variance σ^2. In practice, we use the statsmodels (67) implementation of linear mixed-effect models. Significance of the coefficients were assessed with a t-test, as implemented in statsmodels.

C. Replication across single narratives. To further support that the $\mathcal{R}$ were not driven by the narratives' variability, we replicate the analysis of Figure 1D within single narratives. In Figure S1, we show that correlation scores between brain scores and comprehension scores are positive for each of the seven narratives.

D. Noise Ceiling Estimates. fMRI recordings are inherently noisy. Thus, we estimate an upper bound of the best brain score that can be obtained given the level of noise in the Narrative dataset. To this end, for each (subject, narrative) pair, we linearly map the fMRI recordings, not with the GPT-2 activations, but with the average fMRI recordings of the other subjects who listened to that narrative. More precisely, we use the exact same setting as in Eq. (1), but we predict $Y^{(s)}$, not from $g(X)$ (GPT-2's features after temporal alignment, of size $n_{\text{times}} \times n_{\text{dim}}$), but from the mean of the other subject's brains $\overline{Y} = \frac{1}{|S|}\sum_{s' \neq s} Y^{(s')}$ (of size $n_{\text{times}} \times n_{\text{voxels}}$). This score is called the noise ceiling for the (subject, narrative) pair. The noise ceilings for each brain region are displayed in Figure S2, and correspond to upper bounds of the brain scores displayed in Figure 1B.

E. Replication across the contextual layers of GPT-2. Previous analyses mostly focus on the eight layer of GPT-2. In Figure S4, we compute the brain scores of each layer of GPT-2, and report their

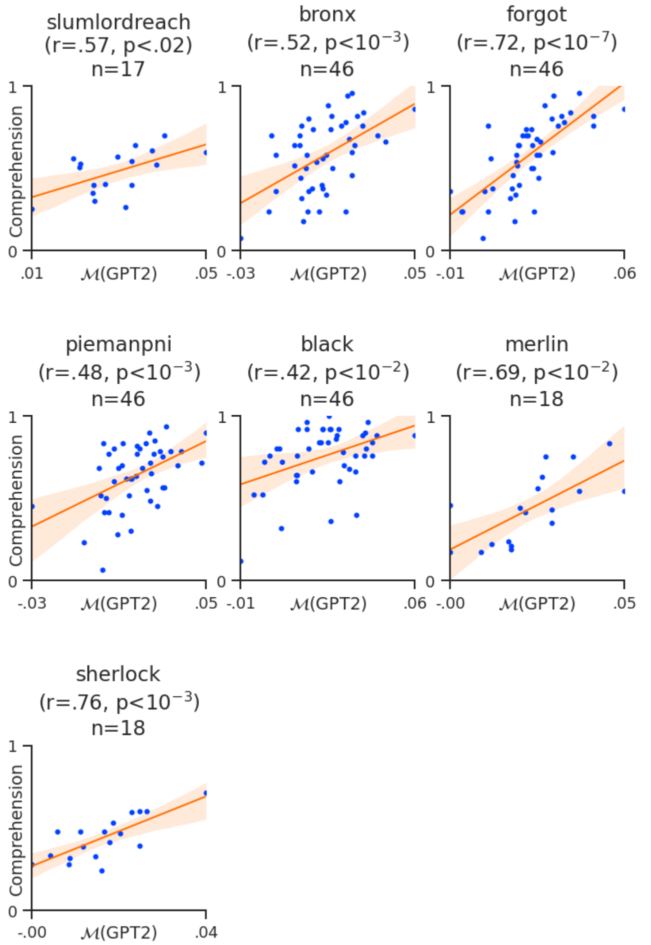

Fig. S1. Replication within single narratives. Same as Figure 1D for each single narrative.

correlation with the subject's comprehension scores. While the correlation with comprehension is the highest in layers 6-to-12 (and thus best explain comprehension's variability), our results do generalize to other contextual layers of GPT-2.

F. Distribution of regularization parameters. To quantify the mapping between the brain signals and GPT-2 activations, we use a ℓ_2-penalized linear regression (cf. Methods). To further investigate how penalization affected the brain score, we compute the optimal regularization parameter alpha for each (subject, narrative, voxel, fold), we average the alphas across (subject, narrative, fold) triplets, and report the corresponding alphas across voxels (left) as well as the relationship between alphas and brain scores (on the right). As shown in Figure S5, regularization parameters are lower in regions commonly associated with language (auditory cortex, supramarginal, inferior-frontal areas) while higher alphas (yellow) are associated with noisier regions.

G. Replication using partial correlation analyses. In Figure 1F, we compute the specific contribution of phonological, lexical and compositional features, respectively. To do so, we favor the simplest and most conservative method by using hierarchical modeling, which consists of computing the brain score of the two sets of features (e.g. Word Embedding vs. Layer 8) and then subtracting the scores. This approach is particularly conservative: the explainable variance shared by two sets of features is by definition fully attributed to the lower-level feature set (i.e. Word Embedding). Thus, our method tends to underestimate the variance specific to deeper layers. The fact that these effects remain largely above chance is thus good evidence that this layer captures representations specifically predictive

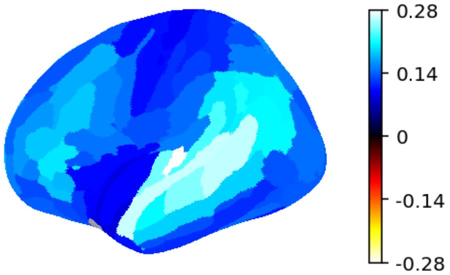

Fig. S2. Noise ceiling estimates. Noise ceilings averaged across subjects, narratives and voxels within each region of interest. They are upper bounds of the brain scores in Figure 1B.

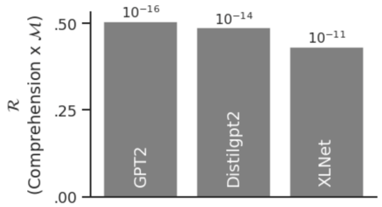

Fig. S3. Replication to two other causal transformer architectures from Huggingface (XLNet base and Distilgpt2). The vertical axis shows the average correlation between (i) comprehension scores and (ii) brain scores. The top text displays the p-values of the corresponding correlation. The mapping scores were averaged across all voxels and the correlation with comprehension was computed, similarly to Figure 1D.

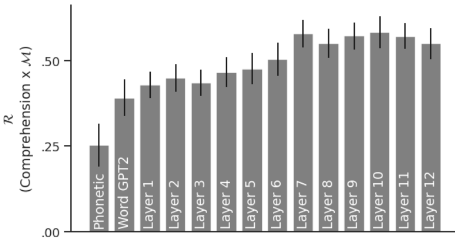

Fig. S4. Correlation between comprehension scores and brain scores, for each layer of GPT-2 as well as phonetic features. Error bars are the standard errors of the means across subjects.

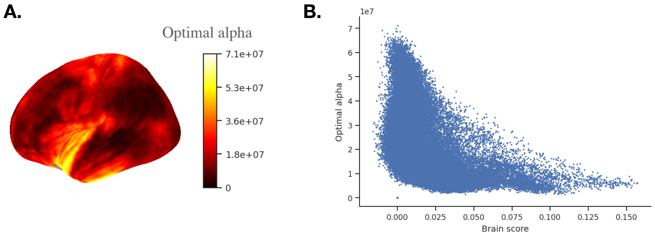

Fig. S5. A) Optimal regularization parameters alpha (log-scaled) across voxels. A penalized regression is fitted for each (subject, narrative, voxel, fold) and the corresponding optimal regularization parameters alphas are extracted. Alphas are averaged across (subject, narrative, fold) to obtain one score per voxel. **B)** the same alphas on the y-axis. On the x-axis, the corresponding brain scores for each (subject, narrative, voxel, fold) averaged across (subject, narrative, fold).

of comprehension.

In Figure S6, we replicate our results with a partial correlation method, i.e. a method that separates two sets of features (Word Embedding and GPT-2) during the fitting of the linear model. Specifically, we fit both Word and GPT-2 models simultaneously with a banded ridge regression (68), and then evaluate the unique variance accounted for by each sub-model. For simplicity, we follow the setup of the original paper (68) and replicate our results for one pair of features (here, Word Embedding and GPT-2). We use the same modeling and cross-validation setting as in Figure 1F. Figure S6 shows the specific brains scores attributed to Word and GPT-2 embeddings, and their specific correlation with comprehension. We obtain similar results as in Figure 1F, but the correlation with comprehension specific to GPT-2 ($R[M''(\text{GPT2})] = 0.52$) is slightly higher than the one in the paper ($R[M'(\text{GPT2}) - M(\text{Word})] = 0.31$).

H. Effect of attention processes in the brain. Is the correlation between comprehension and GPT-2's representations solely due to attentional fluctuation? Indeed, attention can modulate both (i) comprehension and (ii) the average BOLD activity (48, 49) and thus lead to an indirect correlation between these last two variables. To address this issue, we first qualitatively compare our results to those of a meta-analysis covering 6,201 subjects recorded with fMRI during a study related to speech-based or auditory-based attention (Figure S7). The results suggest that these attentional mechanisms are associated with a restricted set of temporal and sensory-motor areas. Furthermore, our analysis of the average BOLD response and its correlation with comprehension highlight a similar cortical network (Figure S8). In both cases, however, these neural bases of attention appear much less distributed than those obtained with GPT-2. In particular, the activations in the prefrontal and parietal cortices as well as in the inferior temporal gyri seem to be specifically accounted for by GPT-2's representations. Overall, while these results call for more direct manipulations of subjects' attention, they suggest that the link between GPT-2 and the brain bases of comprehension is not trivially reducible to attention.

I. fMRI preprocessing. Our analyses rely on the already preprocessed data from Nastase et al. 2020 (61), unsmoothed version. Below, the pre-processing pipeline, as stated in the original paper.

"The functional MRI data were preprocessed in the following way. First, a reference volume and its skull-stripped version were generated using a custom methodology of fMRIPrep. A deformation field to correct for susceptibility distortions was estimated using fMRIPrep's fieldmap-less approach. The deformation field results from co-registering the BOLD reference to the same-subject T1w-reference with its intensity inverted (Huntenburg, 2014; Wang et al., 2017). Registration was performed with antsRegistration (ANTs 2.2.0), and the process was regularized by constraining deformation to be nonzero only along the phase-encoding direction, and modulated with an average fieldmap template (Treiber et al., 2016). Based on the estimated susceptibility distortion, a corrected EPI reference was calculated for more accurate co-registration with the anatomical reference. The BOLD reference was then co-registered to the T1w reference using bbregister (FreeSurfer 6.0.1), which implements boundary-based registration (Greve and Fischl, 2009). Co-registration was configured with six degrees of freedom. Head-motion parameters with respect to the BOLD reference (transformation matrices, and six corresponding rotation and translation parameters) are estimated before any spatiotemporal filtering using mcflirt (FSL 5.0.9; Jenkinson et al., 2002, 2012; Smith et al., 2004). BOLD runs were slice-time corrected using 3dTshift from AFNI (20160207; Cox and Hyde, 1997). The BOLD time-series were resampled onto the following surfaces: fsaverage , fsaverage6 , fsaverage5 . The BOLD time-series (including slice-timing correction when applied) were resampled onto their original, native space by applying a single, composite transform to correct for head-motion and susceptibility distortions. These resampled BOLD time-series are referred to as preprocessed BOLD in original space, or just preprocessed BOLD. The BOLD time-series were resampled into two volumetric standard spaces, correspondingly generating the following spatially-normalized, preprocessed BOLD runs: MNI152NLin2009cAsym , MNI152NLin6Asym . A reference volume and its skull-stripped version were first generated using a custom methodology of fMRIPrep. All resamplings were performed with a single interpolation step by composing all the pertinent transformations (i.e. head-motion transform matrices, susceptibility distortion correction, and co-registrations to anatomical and output spaces). Gridded (volumetric) resamplings were performed using antsApplyTransforms

DRAFT

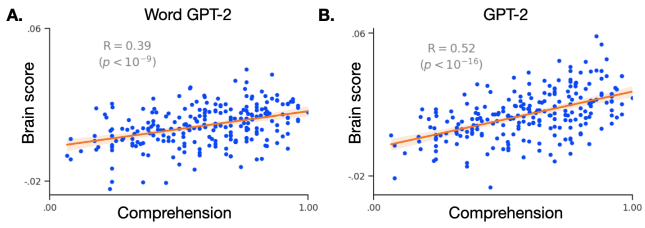

Fig. S6. Same as Figure 1D but using partial correlation analysis: a model is fitted using both Word vectors and GPT-2 as input, we then evaluate the brain score accounted for by each submodel specifically. **A)** Brain scores of the Word vectors specifically, averaged across voxels. **B)** Brain scores of the eight layer of GPT-2 specifically, averaged across voxels. In red, the correlation between comprehension scores (x-axis) and brain scores (y-axis).

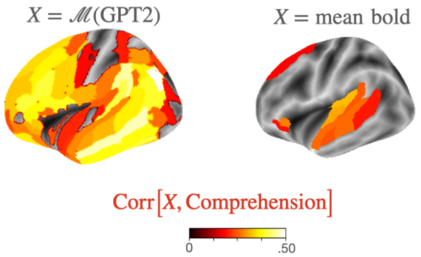

Fig. S8. Correlation between comprehension scores and (a) brain scores of GPT-2 for each (subject, story) pair, (b) the BOLD magnitude, averaged across scans for each subject and story separately.

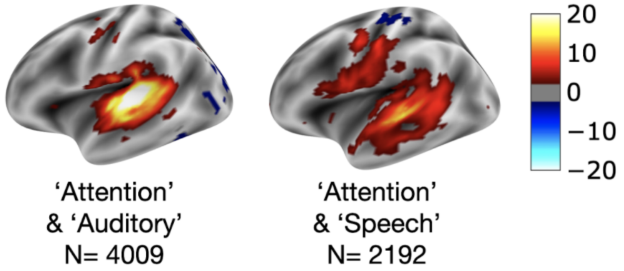

Fig. S7. Meta-analyses from NeuroQuery. Brain networks associated with the concepts of"attentio" combined with "auditory", and "attention" combined with "speech".

(ANTs 2.2.0), configured with Lanczos interpolation to minimize the smoothing effects of other kernels (Lanczos, 1964). Non-gridded (surface) resamplings were performed using mri_vol2surf (FreeSurfer 6.0.1).

Several confounding time-series were calculated based on the preprocessed BOLD: framewise displacement (FD), DVARS, and three region-wise global signals. FD and DVARS are calculated for each functional run, both using their implementations in Nipype (following the definitions by Power et al., 2014). The three global signals are extracted within the CSF, the WM, and the whole-brain masks. Additionally, a set of physiological regressors were extracted to allow for component-based noise correction (CompCor; Behzadi et al., 2007). Principal components are estimated after high-pass filtering the preprocessed BOLD time-series (using a discrete cosine filter with 128s cut-off) for the two CompCor variants: temporal (tCompCor) and anatomical (aCompCor). The tCompCor components are then calculated from the top 5% variable voxels within a mask covering the subcortical regions. This subcortical mask is obtained by heavily eroding the brain mask, which ensures it does not include cortical GM regions. For aCompCor, components are calculated within the intersection of the aforementioned mask and the union of CSF and WM masks calculated in T1w space, after their projection to the native space of each functional run (using the inverse BOLD-to-T1w transformation). Components are also calculated separately within the WM and CSF masks. For each CompCor decomposition, the k components with the largest singular values are retained, such that the retained components' time series are sufficient to explain 50 percent of variance across the nuisance mask (CSF, WM, combined, or temporal). The remaining components are dropped from consideration. The head-motion estimates calculated in the correction step were also placed within the corresponding confounds file. The confound time series derived from head motion estimates and global signals were expanded with the inclusion of temporal derivatives and quadratic terms for each (Satterthwaite et al., 2013). Frames that exceeded a threshold of 0.5 mm FD or 1.5 standardized DVARS were annotated as motion outliers. All of these confound variables are provided with the dataset for researchers to use as they see fit. HTML files with quality control visualizations output by fMRIPrep are available via DataLad.

We next temporally filtered the functional data to mitigate the effects of confounding variables. Unlike traditional task fMRI experiments with a well-defined event structure, the goal of regression was not to estimate regression coefficients for any given experimental conditions; rather, similar to resting-state functional connectivity analysis, the goal of regression was to model nuisance variables, resulting in a "clean" residual time series. However, unlike conventional resting-state paradigms, naturalistic stimuli enable intersubject analyses, which are less sensitive to idiosyncratic noises than within-subject functional connectivity analysis typically used with resting-state data (Simony et al., 2016; Simony and Chang, 2019). With this in mind, we used a modest confound regression model informed by the rich literature on confound regression for resting-state functional connectivity (e.g. Ciric et al., 2017; Parkes et al., 2018). AFNI's 3dTproject was used to regress out the following nuisance variables: six head motion parameters (three translation, three rotation), the first five principal component time series from an eroded CSF and a white matter mask (Behzadi et al., 2007; Muschelli et al., 2014), cosine bases for high-pass filtering (using a discrete cosine filter with cutoff: 128 s, or .0078 Hz), and first- and second-order detrending polynomials. These variables were included in a single regression model to avoid reintroducing artifacts by sequential filtering (Lindquist et al., 2019). The scripts used to perform this regression and the residual time series are provided with this data release. This processing workflow ultimately yields smoothed and non-smoothed versions of the "clean" functional time series data in several volumetric and surface-based standard spaces."

DRAFT
